# Polyuria and Acute Hyperglycemia Secondary to New-Onset Diabetes in a Young Woman With Friedreich’s Ataxia

**DOI:** 10.7759/cureus.16032

**Published:** 2021-06-29

**Authors:** Jasmine Santos, Jason R Woloski, Natasha Wu

**Affiliations:** 1 Family Medicine, Geisinger Health System, Geisinger Commonwealth School of Medicine, Wilkes-Barre, USA

**Keywords:** diabetes, endocrinology and diabetes, friedreich's ataxia, urinary frequency, new-onset diabetes

## Abstract

A 23-year-old woman with progressive Friedreich’s ataxia (FRDA) presented to a local urgent care facility for urinary urgency and frequency. A urinalysis showed the presence of trace ketones and glucose, and point-of-care testing revealed severely elevated glucose. The patient was referred to the emergency department and was admitted for further evaluation of hyperglycemia. Laboratory tests were negative for a urinary tract infection; however, results revealed elevated serum glucose and hemoglobin A1C. She was diagnosed with new-onset diabetes mellitus and started on insulin therapy. Management of her diabetes was complicated due to advanced neurodegenerative symptoms related to FRDA. An individualized treatment plan and coordination of care with her home facility were essential for managing her diabetes.

## Introduction

Friedreich’s ataxia (FRDA) is the most common inherited cause of ataxia, often seen in Caucasian populations. Diabetes mellitus (DM) is a known complication found in up to 30% of patients, which often develops 10-15 years after the diagnosis of FRDA [1]. Annual surveillance for DM is recommended with a fasting glucose test; however, diagnosis is often delayed or revealed after the development of acute symptoms of hyperglycemia. The best practice management of DM in patients with FRDA remains unclear [2].

## Case presentation

We present the case of a 23-year-old Hispanic woman with a past medical history of FRDA and depression. The onset of FRDA was at 13 years of age with initial symptoms of gait instability. By the age of 16, she had progressive ataxia which required her to become wheelchair-bound. She now resides in a group home for assistance with activities of daily life due to incomplete quadriplegia. She has an older brother and younger sister with FRDA who were similarly diagnosed in their early teens.

In September 2020, our patient presented to a local urgent care facility for dysuria and urinary frequency. A urinalysis was performed that indicated trace ketones and 3+ glucose, along with negative protein, nitrite, and esterase. Point-of-care glucose testing was completed and found to be high (>500 mg/dL). She was referred to the emergency department (ED) and admitted to our hospital service for further evaluation of hyperglycemia. All previous urine studies were negative for glucose and ketones, including those conducted only a few months prior to this presentation.

A review of systems revealed dry mouth, increased thirst, polyuria, and polydipsia. Physical examination showed mild dysarthria, bilateral horizontal nystagmus, dysmetria, scoliosis, uncoordinated limb movements in all four extremities, and ataxic gait. On admission, her vitals were stable, and her body mass index (BMI) was 32. A repeat urinalysis in the ED confirmed trace ketones and >1,000 mg/dL glucose in the urine. Laboratory results revealed serum glucose of 582 mg/dL with an anion gap of 14. Further studies revealed hemoglobin A1c of 10.6% with estimated average glucose of 258 mg/dL. The C-peptide level was 1.8 mg/mL (0.8-4.2 ng/mL). Anti-islet cell and GAD65 antibody assays were negative.

Our patient’s normal C-peptide level, negative anti-islet and GAD65 antibody results, lack of ketoacidosis, along with a BMI of >30 led us to rule out an autoimmune etiology such as type 1 diabetes and latent autoimmune diabetes in adults in favor of a type 2 diabetes diagnosis. The patient was previously insulin-naive. During her inpatient stay, she was started on a combination of long-acting and short-acting insulin along with carb coverage at a ratio of 1:15. Our inpatient diabetes educator was consulted and glucose levels responded appropriately to treatment.

Given the social complexity of the case, prior to discharge, the insulin treatment regimen was adjusted. To minimize demand on facility staff and maximize patient comfort, she was discharged with once-daily, long-acting injectable insulin along with oral metformin and repaglinide. A high-risk outpatient endocrinology appointment was scheduled.

With support from the group home, our patient remained compliant with her medications; however, her daily blood sugar levels remained above 300 mg/dL. Two days after discharge, she returned to the ED for hyperglycemia. Labs were obtained. Her serum glucose was 390 mg/dL with no associated symptoms. Diabetic ketoacidosis was ruled out and she was treated with subcutaneous fast-acting insulin prior to discharge.

The patient’s follow-up care consisted of a combination of in-person and telemedicine visits with a clinical pharmacist, an endocrinologist, and her family practitioner. She experienced side effects of mild gastrointestinal upset and diarrhea, likely from oral pharmacologic agents. She also experienced lower extremity edema. She was given furosemide as needed and a referral to cardiology for further evaluation. She also received a referral to see a diabetes dietician.

Over the course of five weeks, our patient’s diabetes medication regimen was adjusted until glucose levels stabilized and side effects minimized. Her final regimen included oral extended-release metformin twice daily, once-daily long-acting insulin glargine, and fast-acting insulin aspart three times daily with meals. Lastly, within two months of discharge, the patient was diagnosed with an acute right lower extremity deep venous thrombosis and started on anticoagulation with apixaban.

## Discussion

FRDA is the most common inherited cause of ataxia with prominent non-neurological causes of morbidity including cardiomyopathy and diabetes [1]. The mechanism of injury is driven by a mutation in the *frataxin* gene which is associated with mitochondrial iron dysregulation, altered lipid synthesis, and an increase in reactive oxygen species [3]. Various models have shown that this oxidative stress leads to cell apoptosis and is thought to be the source of beta-cell destruction and loss of glucose tolerance in patients with FRDA [3,4]. According to one study, reduction in beta-cell mass leads to insulin deficiency and development of type 2 diabetes without autoimmune involvement [4].

Patients with FRDA may experience impaired glucose tolerance and are at an increased risk of developing diabetes [5]. The diagnosis is often made after the development of acute symptoms such as polyuria and polydipsia, or the discovery of ketoacidosis [5-7]. Urological symptoms such as hesitancy, urgency, and incontinence are common in FRDA and can mimic polyuria or symptoms of a urinary tract infection [1]. A thorough history and review of systems along with high clinical suspicion for diabetes in this patient population are vital for diagnosis.

It is critical to identify diabetes in these patients and start treatment without delay. In a case series, two patients with FRDA had dramatic worsening of their cardiac function after the development of endocrinopathies. One patient with diabetic ketoacidosis had rapid progression of severe left ventricular dysfunction [7]. In another case series, a male with FRDA was diagnosed with diabetes after presenting with polyuria and polydipsia without ketoacidosis. His BMI was 31 and hemoglobin A1c 9.8%. In his case, as he grew older, he became noncompliant with his diabetes care due to depressive symptoms. He eventually developed severe ketoacidosis which was complicated by a diabetic coma and cardiac arrest [5]. Patients should be regularly assessed for insulin-dependent DM to prevent such critical complications.

Surveillance for diabetes in patients with FRDA is recommended annually with a fasting glucose test [8]. However, diabetes remains underdiagnosed in this population using solely fasting glucose tests [9]. Several studies have found that an oral glucose tolerance test (OGTT) is a more accurate diagnostic tool to capture patients with FRDA at risk for glucose intolerance and diabetes [9-11]. Utilization of OGTT at annual wellness visits may lead to earlier diagnosis and intervention, leading to decreased acute events and improved clinical outcomes.

As patients with FRDA have both insulin deficiency and insulin resistance, treatment of type 2 diabetes in these patients should include interventions addressing both these issues [4,10]. Lifestyle modifications to increase physical activity should be encouraged to decrease adiposity over time. Pharmacological therapies such as metformin or thiazolidinedione can be used to improve insulin sensitivity. However, we caution against the use of these drugs in this population. There are noted indirect effects on mitochondria given the mechanism of action that inhibits complex I of the mitochondrial respiratory chain, which may compound the existing disease pathology in FRDA [4,7]. In addition, thiazolidinediones have been associated with congestive heart failure and increased adipose tissue, which may pose additional risks to these patients [4,7]. Adding other medications such as sulfonylureas, glucagon-like peptide 1 (GLP-1) analogs, and dipeptidyl peptidase IV inhibitors to the treatment plan are an option to stimulate insulin secretion. There is evidence that GLP-1 agents may have beta-cell and neuroprotective properties; however, this is yet to be confirmed in comparative controlled trials [11]. Despite the advancement in oral hypoglycemics, exogenous insulin remains the most effective treatment for diabetes [2,4].

Diabetes is independently associated with worse functional status among patients with FRDA [12]. Given this, many patients have a reduced ability to perform activities of daily living (ADLs) and adhere to medication without assistance. In a case study, two siblings over the age of 50 with a medical history significant for FRDA and depression were chronically institutionalized for incomplete quadriplegia, musculoskeletal deformities, hypertrophic cardiomyopathy, and diabetes. Both were wheelchair-bound requiring assistance with ADLs. The authors found that the management of spasticity, neuropathic pain, and the symptomatic treatment of heart failure and diabetes with the help of nursing care improved the quality of life of these patients [12]. This emphasizes the importance of support systems and coordinated care for patients with FRDA [6,12]. Individualized treatment regimens with insulin, with or without antidiabetic medications, are crucial for the successful control of diabetes in these patients [6]. Compliance with insulin is vital as poor adherence can lead to severe adverse outcomes as mentioned above.

In this case, we identified success in our patient’s care management as well as opportunities for improvement. Our inpatient team quickly diagnosed diabetes based on the presenting signs and symptoms. Promptly involving the diabetes educator and case management team ensured that our patient returned safely home to her group facility with the preliminary equipment and education needed to manage her new diabetes diagnosis. Unfortunately, the once-daily dosing was not sufficient in controlling her glucose and she returned to the ED a few days after discharge.

In retrospect, additional training for the group home staff regarding the management of elevated blood sugar and the use of the on-call provider may have prevented this return ED visit. In addition, emphasized instructions to call the primary care provider with questions along with a closer follow-up, perhaps within 24-72 hours after the initial discharge, may have been another way to prevent the repeat ED visit. Additionally, the development of lower extremity edema shortly after the initiation of insulin was concerning. The etiology may have been multifactorial including copious intravenous fluids during the hospital visit, decreased mobility, and recent initiation of insulin where peripheral edema is a noted common reaction to long-acting insulin formulations. We also must consider the development of cardiomyopathy which is a common comorbidity for individuals with FRDA. However, in our patient, a subsequent echocardiogram was unremarkable. Lastly, a new unilateral deep venous thrombosis was discovered approximately two months after the initial hospitalization. The etiology was complex, given chronic baseline mobility challenges complicated by acute worsening from new bilateral lower extremity edema and the recent hospitalization.

Consistent follow-up visits with her care team since then have prevented any additional emergency visits and have helped ascertain the ideal medication regimen to manage her diabetes at home. Overall, an individualized treatment plan and coordination of care with her group home facility were essential for her diabetes management and, most importantly, allowed her to preserve her quality of life.

## Conclusions

Diabetes is a common complication in patients with FRDA and should be routinely screened for by healthcare providers, preferably via an OGTT. Treatment of diabetes can be challenging due to neurodegenerative symptoms that may interfere with the ability to self-administer insulin. Additionally, close follow-up with cardiology is important to monitor for any signs or symptoms of cardiomyopathy, especially after the initiation of diabetes medications. Once diabetes is diagnosed, an individualized treatment plan along with efficient coordination of care is essential for successful diabetes management in patients with FRDA.
